# Addressing Nursing Home Residents’ Goals, Preferences, and Priority: A Local and National Imperative

**DOI:** 10.1093/geroni/igaf122.1985

**Published:** 2025-12-31

**Authors:** Laci Cornelison, Addison Van Zutphen

**Affiliations:** Kansas State University, Manhattan, Kansas, United States; Kansas State University Center on Aging, Manhattan, Kansas, United States

## Abstract

The regulatory F-tag F656—Comprehensive Care Plans—is consistently among the top ten most cited deficiencies in nursing home surveys, highlighting a widespread challenge: nursing homes often lack the resources or processes to meaningfully address residents’ goals, preferences, and priorities (GPPs). To address this gap, we developed a practical, user-friendly guide to help nursing home staff engage residents in conversations about what matters most to them and integrate those insights into care plans and ultimately into daily care provided. In partnership with the Kansas State University Center on Aging, we are piloting this guide and process in up to 30 nursing homes. The pilot includes education on goal-concordant care, structured action planning for individualized quality improvement (QI) projects, and evaluation of implementation progress. Findings from this pilot will inform revisions to the guide and underlying process to prepare for broader dissemination. In addition to improving person-centered care at the nursing home level, we share preliminary lessons that could inform the design of health information technology (HIT) systems capable of capturing and exchanging resident GPPs across care settings. We also consider how structured approaches to GPPs could contribute to the development of goal-concordance measures with relevance for federal regulation and quality monitoring. This paper shares the design, implementation strategy, and early insights from this ongoing initiative, along with its broader implications for systems change in long-term care.

